# Case report: optimal tumor cytoreduction and octreotide with durable disease control in a patient with MEN-1 and Zollinger-Ellison syndrome—over a decade of follow-up

**DOI:** 10.1186/s12957-019-1758-6

**Published:** 2019-12-09

**Authors:** Lynsey M. Daniels, Marian Khalili, William F. Morano, Michaela Simoncini, Beth C. Mapow, Andrea Leaf, Wilbur B. Bowne

**Affiliations:** 1 Department of Surgery, Drexel University College of Medicine, 245 N. 15th Street, Suite 7150, Philadelphia, PA 19102 USA; 2 Department of Medical Oncology, New York Harbor VA Medical Center, Brooklyn, NY USA; 3 Department of Pathology, Drexel University College of Medicine, Philadelphia, PA USA

**Keywords:** Zollinger-Ellison syndrome, Multiple endocrine neoplasia type 1, Octreotide, Gastrinoma, Cytoreduction, Carcinoid tumors, Pancreaticoduodenectomy, Gastrectomy

## Abstract

**Background:**

Zollinger-Ellison syndrome (ZES) is a rare condition characterized by hypersecretion of gastrin by gastrinoma tumors leading to severe peptic ulcer disease with potential development of gastric carcinoid tumors. Herein, we report the clinical course of a 68-year-old patient with multiple endocrine neoplasia type 1 (MEN-1) who underwent several surgeries to ultimately undergo optimal tumor cytoreduction of locally advanced gastrinomas and symptomatic gastric carcinoids. The patient was subsequently maintained on octreotide long-acting release (LAR). This case report supports consideration for aggressive tumor cytoreduction and octreotide in similar patients with MEN-1-associated ZES for durable disease control and symptom management.

**Case presentation:**

The patient is a 68-year-old male with multiple endocrine neoplasia type 1 (MEN-1), diagnosed in 1993 after presenting with recurrent renal calculi and hypercalcemia. Soon thereafter, he presented with symptoms and elevated gastrin levels suggestive of ZES prompting abdominal exploration with partial resection of the duodenum to remove gastrinoma tumor nodules. Within 4 years of the operation, he represented with intractable hypergastrinemia despite optimal medical management with peak gastrin levels exceeding 29,000 pg/mL, in 2006. In January 2007, the patient returned to the operating room for resection of regional peripancreatic and perigastric lymph nodes and enucleation of pancreatic body and tail gastrinoma tumors. Although his gastrin level decreased to 5000 pg/mL with resultant improvement of symptoms, in less than 2 years, he developed disease progression with obstructive symptomatology from enlarging gastric carcinoids and rising gastrin levels. In May of 2008, he underwent pancreaticoduodenectomy and near-total gastrectomy. Since June of 2008, the patient shows no demonstrable progression of disease and remains asymptomatic on LAR octreotide (30 mgs). Gastrin levels have been well controlled (range, 100–624 pg/mL; current 114 pg/mL).

**Conclusion:**

Success of this procedure in our case report highlights the potential role for optimal tumor cytoreduction and LAR octreotide to control disease progression in a patient with MEN-I and Zollinger-Ellison syndrome with locally advanced gastrinoma and secondary large gastric carcinoids.

## Background

Gastrinomas occur in one to three cases per million people in the USA annually, making them the second most common functional, pancreatic neuroendocrine tumor (PNET) [1]. Zollinger-Ellison syndrome (ZES) results from hypersecretion of gastrin producing gastrinoma tumors by which overproduction of gastric acid leads to severe peptic ulcer disease and the possible development of gastric carcinoid tumors. Hypergastrinemic states may lead to type 1 (from atrophic gastritis or pernicious anemia) and type 2 (from ZES) gastric carcinoid tumors [1, 2]. Type 2 gastric carcinoids most commonly develop in patients with ZES due to multiple endocrine neoplasia-1 (MEN-1), despite 80% of ZES cases occurring sporadically [2, 3].

ZES typically presents with signs and symptoms of acid hypersecretion such as peptic ulcer disease (PUD), gastroesophageal reflux disease (GERD), and secretory diarrhea. While a majority of ZES occurs sporadically, approximately 20% of patients with ZES have a familial form associated with multiple endocrine neoplasia type 1 (MEN-1). MEN-1 should be excluded in a patient who presents with ZES as nearly 50% of patients with MEN-1 will develop ZES. Clinical clues to concurrent MEN-1 diagnosis is a family history of PUD as well as personal history of nephrolithiasis and/or hypercalcemia, fasting hypoglycemia, secretory diarrhea, pituitary tumors, parthyroid adenomas and adrenocortical tumors [3].

## Case presentation

**Fig. 1:**
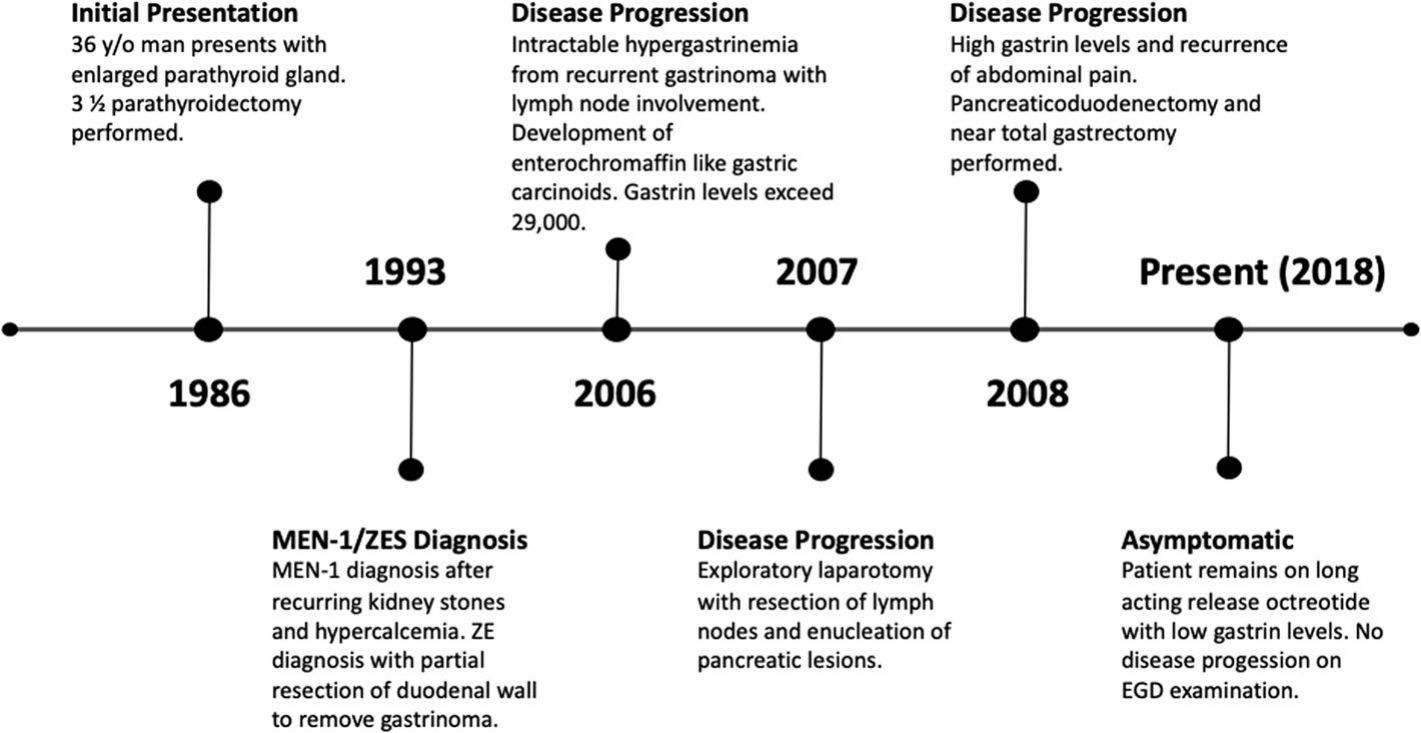
Patient timeline. Patient timeline showing initial presentation in 1986 and subsequent multiple endocrine neoplasia type 1 (MEN-1) and Zollinger-Ellison syndrome (ZES) diagnosis in 1993. Progression of disease detailed throughout the 2000s with accompanying surgical procedures. Patient continues to be asymptomatic at the present date with no disease progression seen on repeat imaging and endoscopy with current gastrin level 114 pg/mL in 2018

**Fig. 2:**
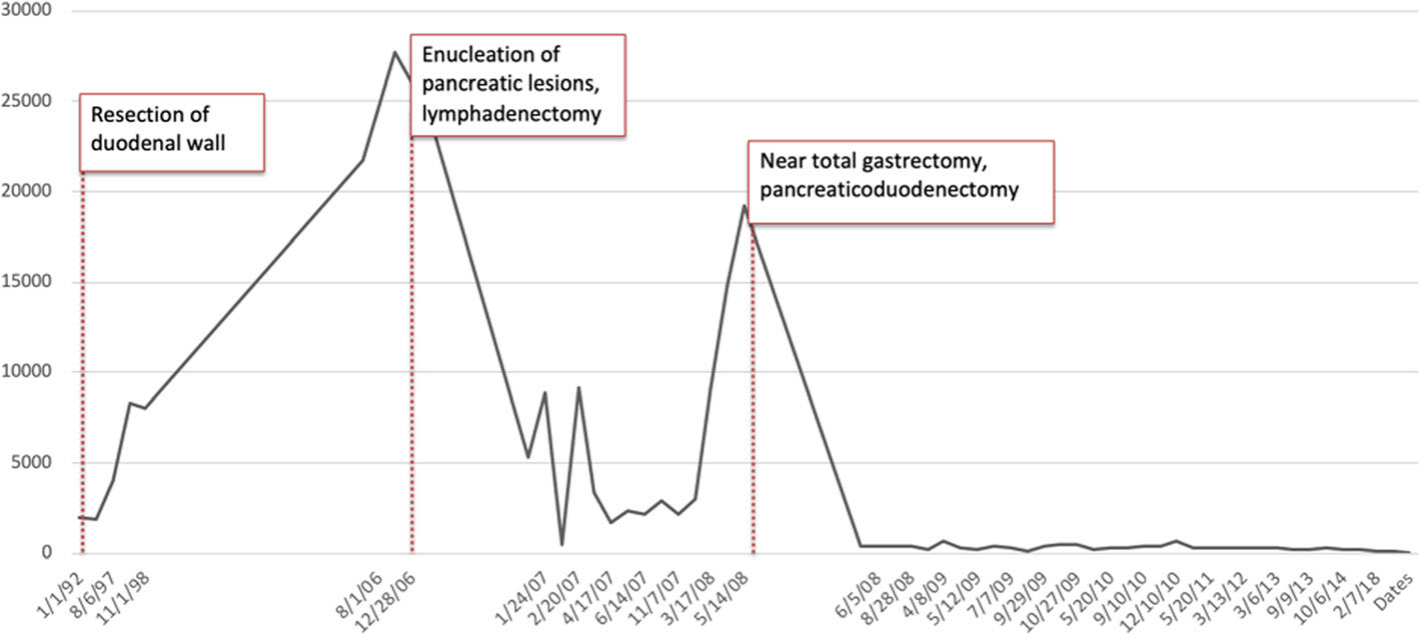
Gastrin levels (pg/mL) over time with noted dates of relevant surgical procedures. Gastrin levels reached a level of 1925 pg/mL prior to excision of duodenal gastrinoma. Gastrin levels then peaked at 27,667 pg/mL in 2006 prior to enucleation of pancreatic lesions. In 2008, gastrin levels again rose to 19,262 pg/mL prior to near-total gastrectomy and pancreaticoduodenectomy. After this procedure, the patient was started on long-acting release octreotide. From 2008 until the most recent gastrin level obtained in 2018, gastrin level was 114 pg/mL

The patient is a 68-year-old man with a history of multiple endocrine neoplasia type 1 (MEN-1), initially diagnosed in 1993 when he was found to have symptomatic calcium nephrolithiasis secondary to hypercalcemia (Fig. 1). Premorbid, the patient exhibited an optimal performance status with normal nutritional parameters. Medical history was non-contributory aside from pharmacologically well-managed schizoaffective disorder. The patient was previously employed in the airline and postal service industries. Although family history was non-contributory, genetic testing confirmed presence of a mutation in the MEN1 gene. He subsequently underwent total thyroid and parathyroidectomy with parathyroid re-implantation in his left forearm. Later in 1993, he began to complain of diarrhea and was discovered to have elevated gastrin levels (Fig. 2) consistent with Zollinger-Ellison syndrome, prompting abdominal exploration with duodenectomy and resection of gastrinoma tumors.

After this initial operative procedure, the patient was placed on omeprazole (20 mg BID). His symptoms were well controlled initially, but in 4 years began to complain of vague upper abdominal symptoms such as nocturnal “gurgling” and “uneasiness,” partially relieved by antacids. His fasting serum gastrin levels (pg/mL) were found to be steadily increasing over a 9-month interval period (4000 pg/ml, August 6, 1997; 8288 pg/ml, April 1, 1998). Despite an increase in his prescribed dose of omeprazole (40 mg BID), symptoms persisted. A somatostatin receptor scintigraphy (SRS) scan was performed showing 2 small fossae of disease near the second portion of the duodenum.

Subsequent upper endoscopies in 2003 and 2006 demonstrated disease progression with innumerable polyps in the stomach (cardia, fundus, body, and antrum) and duodenum (duodenal bulb and 2nd portion). Pathologic diagnosis of gastric carcinoid tumors and duodenal gastrinomas was confirmed. His gastrin levels continued to rise despite medical management, with a maximum gastrin level of 29,000 pg/mL, in 2006.

In late 2006, he underwent exploratory laparotomy, enucleation of pancreatic body/tail lesions, and peripancreatic, periduodenal, and perigastric lymphadenectomy. Surgical pathology showed metastatic neuroendocrine carcinoma, which was gastrin- and chromogranin-positive, compatible with metastatic gastrinoma in the lymph nodes and pancreas. The patient’s postoperative gastrin level subsequently decreased to 5000 pg/mL. He was started on octreotide LAR 20 mgs  Q28d. To control gastrin levels, his prescribed dose was increased to 30 mgs. He has been maintained on this dose to-date.

**Fig. 3:**
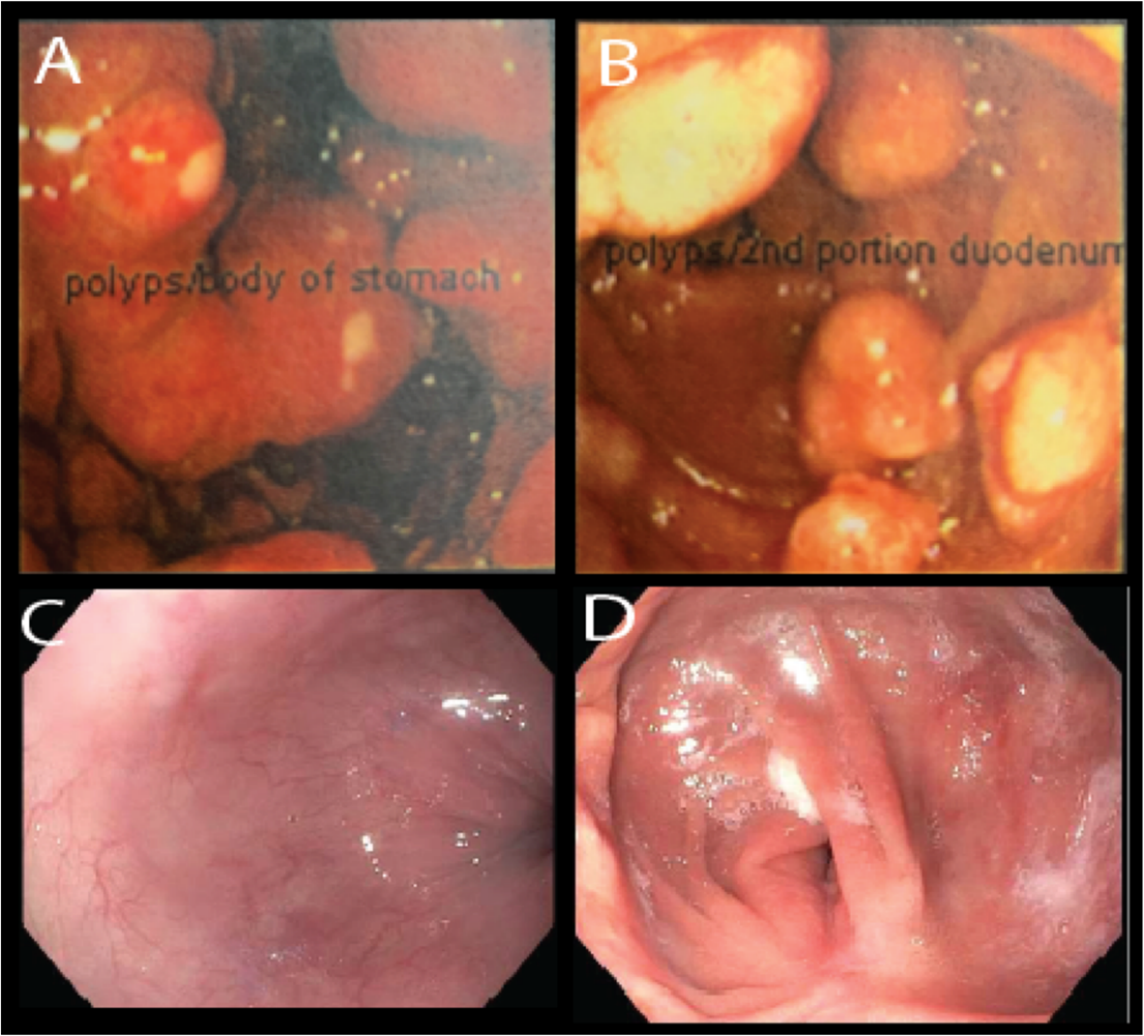
**a** Multiple, large, nearly obstructing ECL-like carcinoid tumors seen in the stomach from endoscopy in 2007. **b** Large gastrinomas in proximal duodenum in 2007. **c** Proximal stomach devoid of ECL-like gastric carcinoid tumors from follow-up in 2018. **d** Distal remnant stomach and small bowel anastomosis devoid of gastric carcinoid tumors on follow-up in 2018

A follow-up endoscopy in late 2007 was significant for disease progression with enlarging multiple, nearly obstructing, polypoid-appearing masses with superficial ulcerations in the stomach (cardia, fundus, proximal antrum) and duodenum (bulb and proximal second portion) (Fig. 3a, b). A series of gastrin levels taken during 2007 ranged from 497 to 9178 pg/mL.

**Fig. 4:**
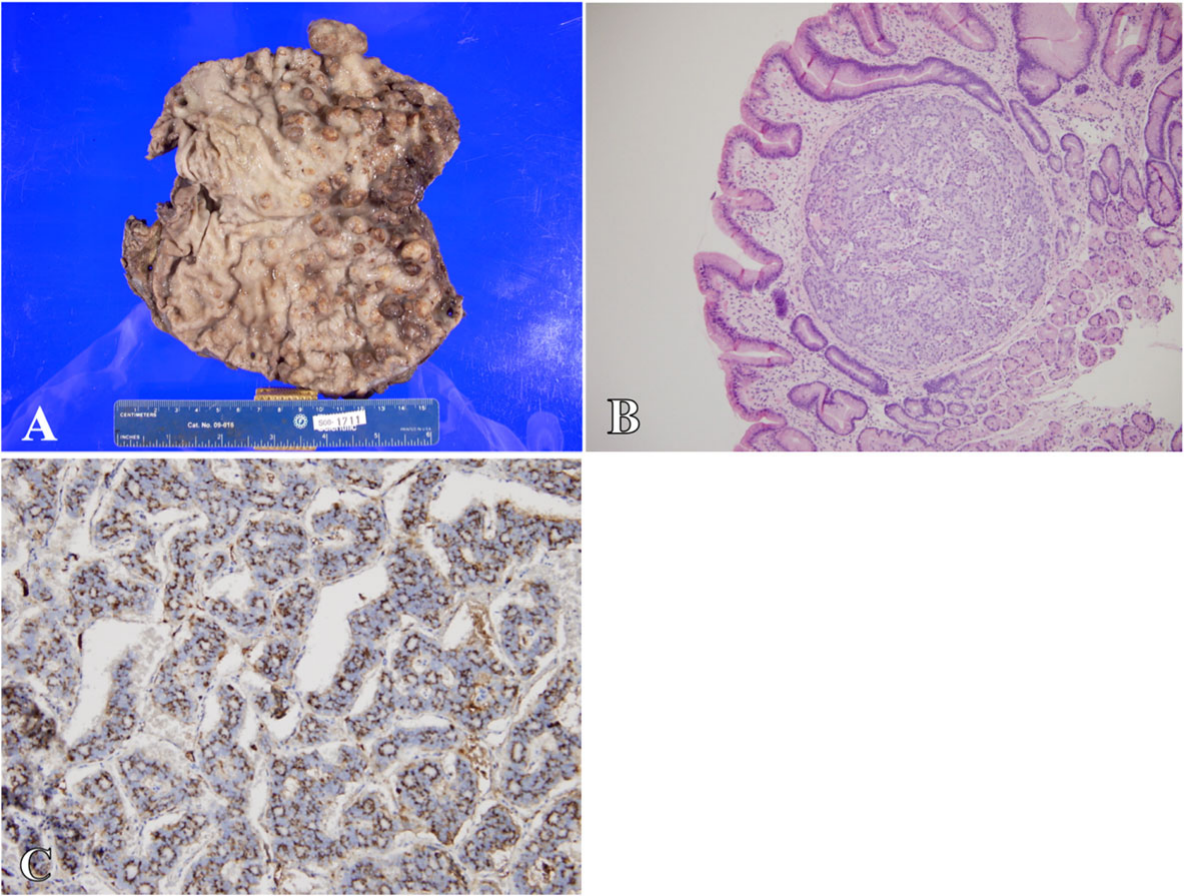
**a** Gross section of stomach showing widespread multifocal neuroendocrine tumor with **b** × 10 magnification hemotoxylin and eosin staining with nest of neuroendocrine tumor cells and **c** × 20 magnification showing immunohistochemical staining chromogranin-positive, gastrin-negative staining consistent with carcinoid tumors

**Fig. 5:**
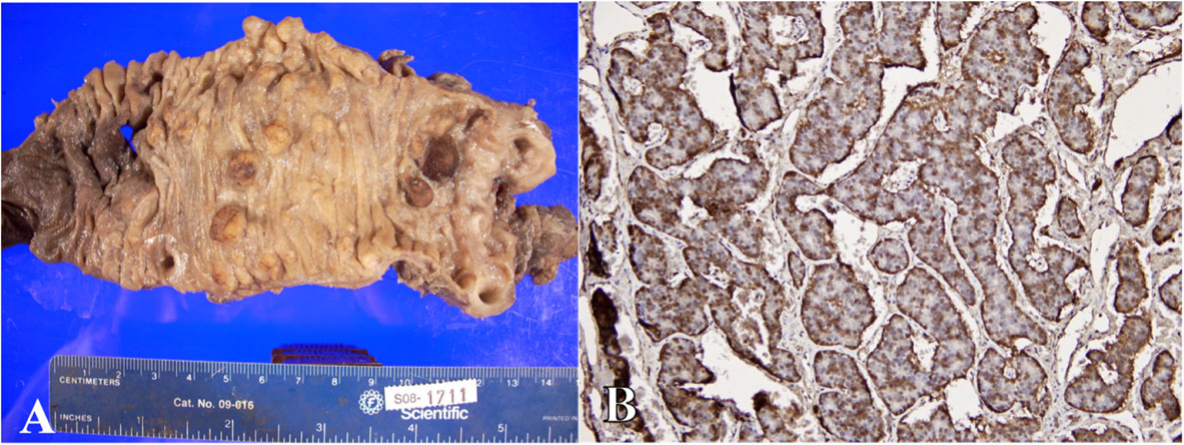
**a** Gross section showing portion of duodenum with **b** × 20 magnification immunohistochemistry showing gastrin-positive staining consistent with gastrinoma

In early 2008, the gastrin levels continued to increase despite octreotide LAR therapy (January, 2994 pg/ml; March, 9084 pg/ml; April, 14,813 pg/ml; May. 19,262 pg/mL). After a multi-disciplinary group convened, the decision was made to perform optimal tumor cytoreduction. In May 2008, the patient underwent a pancreaticoduodenectomy with near-total gastrectomy. Surgical pathology of the stomach indicated widespread multifocal neuroendocrine tumor invading into the lymphatics, subserosa, and perigastric lymph nodes; tumors were found to be chromogranin-positive and gastrin-negative consistent with carcinoid tumors (Fig. 4). Multiple duodenal tumors and pancreatic head lesions were gastrin- and chromogranin-positive, consistent with gastrinoma (Fig. 5).

Since the optimal cytoreductive surgical procedure in 2008, the patient has remained on octreotide LAR (30 mgs Q28d) and esomeprazole (20 mg BID). His gastrin levels have been well controlled (range, 100–624 pg/mL; current 114 pg/mL) with no radiographic evidence of disease progression or progression of gastric carcinoids on surveillance endoscopy (Fig. 3c, d). Normalization of serotonin and chromogranin A levels were observed (data not shown)

## Discussion and conclusions

Management of patients with sporadic ZES differs from management of MEN-1-associated ZES. This is, in part, due to higher rates of enterochromaffin like (ECL) tumors in MEN-1 compared to sporadic ZES (30% versus 5%), natural history of each condition, and probability of cure [4, 5]. Although it is known that surgery plays a key role in the treatment of ZES, timing of surgery and extent of resection remains controversial [5]. This discussion will address historical considerations in ZES management, current evidence-based recommendations, and the experience gained from our patient.

Zollinger-Ellison syndrome was defined in 1956 by Drs. Robert M. Zollinger and Edwin H. Ellison. They described two patients who had developed recurrent and multifocal ulcerative lesions in the GI tract in addition to associated tumors involving the pancreas. The clinical triad they described included mucosal ulcerations, acid hypersecretion, and presence of non-β-cell pancreatic tumors [5]. By the 1960s, gastrin was discovered to be the hormone responsible for gastric hypersecretion and secretin stimulation was known to play a role in gastrin levels [5].

Although only a small percentage of patients with PUD are diagnosed with ZES (and only about 20% of ZES cases are MEN-1-associated), it is widely accepted that any patient with ZES should be screened for MEN-1 [3, 5]. The initial screen should include a serum calcium and parathyroid hormone study. Detection of primary hyperparathyroidism (PHPT) indicates the patient has MEN-1. Surgery for PHPT should precede any procedures for gastrinoma. In these patients, endoscopic gastroduodenoscopy should be performed to evaluate for mucosal disease. Prior to surgery, radiologic imaging identifies patients whom may benefit from surgery. While there have been significant improvements in CT and MRI technology, it is unlikely that these imaging modalities will contribute to comprehensive detection of gastrinoma tumors [5, 6]. Because of higher sensitivity and specificity, somatostatin receptor scintigraphy (SRS), otherwise known as octreoscan, is the preferred study to detect primary tumors and metastatic lesions in ZES [5].

Historically, prior to the availability of histamine-2 receptor blockers and PPIs, acid-reducing surgery was necessary to control acid hypersecretion in ZES [3]. However, today, PPIs are a mainstay of ZES therapy, which is attributed to their long-lasting efficacy and oral availability. In our patient, a PPI was initially used to control symptoms; however, even after maximal medical therapy, the patient continued to experience symptoms. Although PPIs may control symptomatology in most patients, endoscopic surveillance is still recommended with routine screening at regular intervals as mucosal healing does not necessarily correlate with symptom relief [5, 7].

Long-acting somatostatin analogs (SSAs) have been proposed as therapeutic treatment for malignant gastrinomas that are deemed not surgically resectable [8]. There have been two important phase III randomized trials, PROMID and CLARINET that, in part, evaluate the role of SSAs in control of tumor progression [9, 10]. As with our patient, those with hypergastrinemia are typically placed on an SSA as a means of controlling gastrin production.

The PROMID study investigated the antitumor effect of octreotide LAR in patients with locally inoperable or metastatic neuroendocrine tumors (NETs) [10]. However, patients with gastrinomas, ZES, or primary pancreatic (p) or duodenal NETs were not included in this study. Although PROMID demonstrated that octreotide LAR significantly prolonged time to disease progression, this study is not directly applicable to our patient [10]. The CLARINET study randomized patients with pNETs to receive either lanreotide or placebo [9]. The results of this study suggested that lanreotide may be effective for the treatment of pNETs. The number of gastrinoma patients included in this study were small; thus, conclusions about efficacy of lanreotide in this patient population is limited [9]. Currently, recent international guidelines (including ENETs 2016, NCCN 2-2018, and NANETs 2013) do not provide specific indications for SSA therapy in ZES NETs. There have been few studies exploring SSA treatment in patients with MEN-1. The first of these reports, performed in 1988, included a single patient treated with long-acting somatostatin which demonstrated efficacy in the management of paraneoplastic ACTH and LDH overproduction in a gastrinoma/MEN-1 patient [11]. In a subsequent study from 2002, 3 MEN-1 patients and 12 with sporadic ZES were initially treated with octreotide 200 μg q12hr followed by maintenance LAR octreotide 20-30 mgs. Unfortunately, individual results for the MEN-1 patients could not be extrapolated, although 53% of the patients had an objective treatment response (stabilization and/or decrease in tumor size) [12]. In 2000, another study investigated the use of long-term treatment of LAR somatostatin analog (SSA) in three patients with MEN-1-associated ZES and carcinoid tumors [13]. In all three patients, EGD showed reduced number and size of carcinoid tumors after 6 months of therapy and gross resolution of disease in 1 year [13]. While these patients had numerous tumors, all patients in this study had small tumors < 1 cm in size, suggesting that LAR SSA may be beneficial in controlling smaller disease burden [13]. Importantly, in similar studies, even with small sample sizes most patients demonstrated both an improvement in symptoms and stabilization of disease [2]. Although there have been few reports looking at SSAs in ZES patients, in our patient, long-acting SSA appeared contributory in controlling disease progression after optimal tumor cytoreduction.

**Fig. 6:**
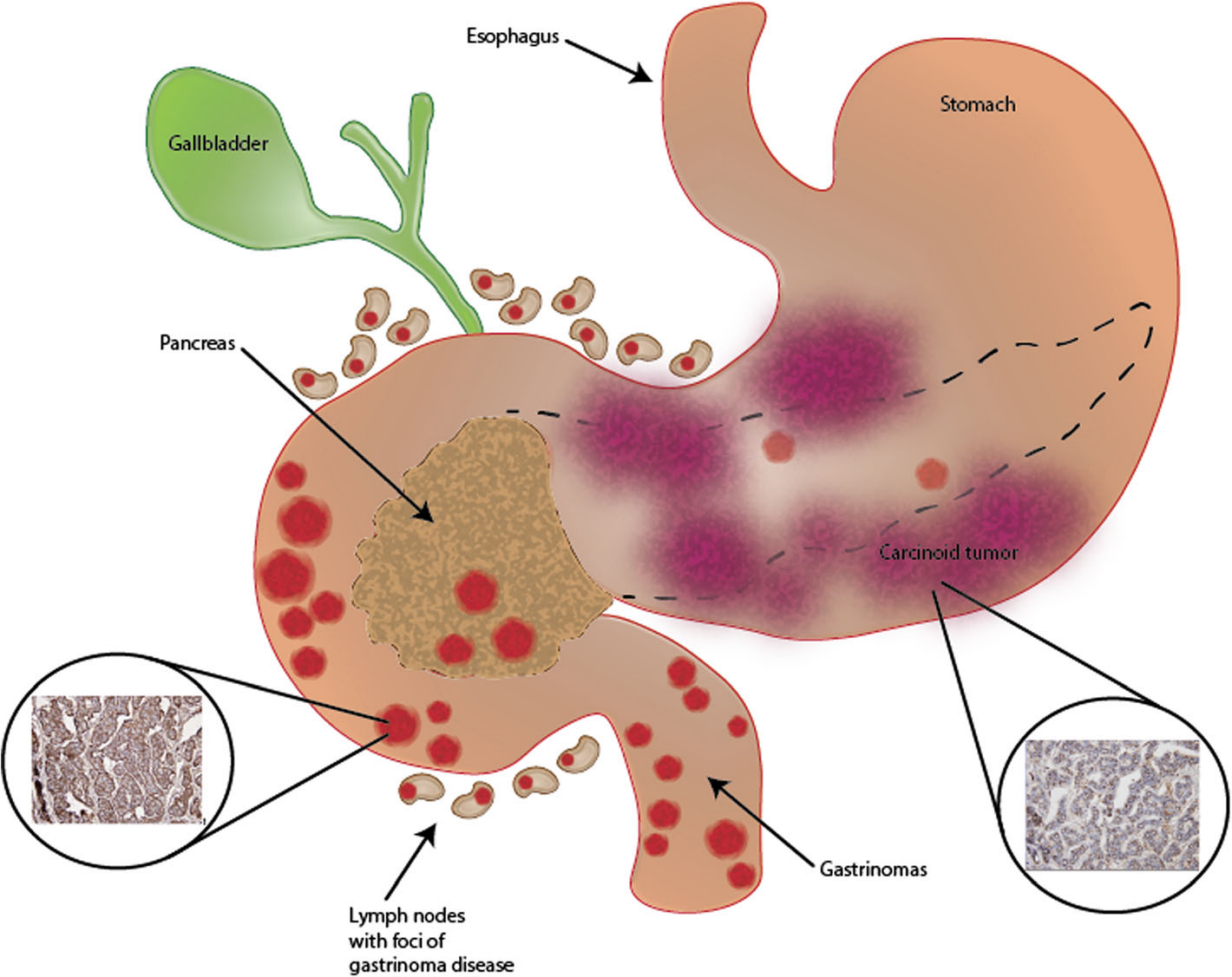
Zollinger-Ellison syndrome (ZES) is caused by gastrin secreting tumors that stimulate the acid-secreting cells of the stomach to maximal activity, resulting in subsequent gastrointestinal mucosal ulceration. The primary tumor of ZES, gastrinomas, frequently occurs within the gastrinoma triangle and result in development of secondary carcinoids from hypergastrinemia. In this patient, clinically depicted in this image, nearly obstructive gastric carcinoids causing gastric outlet symptomatology were present. Additionally, multiple, large tumor nodules of gastrinomas were present in the duodenum with lymph nodes containing foci of gastrinoma disease

The clinical scenario in our patient is depicted in Fig. 6. Briefly, gastric carcinoids are derived primarily from histamine-secreting enterochromaffin-like (ECL) cells. ECL cells, located in the gastric oxyntic region, are in contact with chief cells and parietal cells, albeit there is higher ratio of contact with chief cells [14]. ZES-associated carcinoids (type 2 lesions) are typically small, multiple, located in the fundus and antrum of the stomach and arise from gastrin hypersecretion, leading to gastric and duodenal ulcers at diagnosis [8, 15, 16]. Gastrin, secreted from antral G-cells, binds to cholecystokinin (CCK)-B receptors located on ECL cells membranes leading to histamine release and gastric acid secretion [15]. Gastrin overproduction from locally advanced ectopically producing gastrinoma tumors as shown in Fig. 6 are the ultimate drivers of Type 2 ECL-like carcinoid tumor progression [15]. In the majority of patients with MEN-1-associated ZES (75%), gastric carcinoid tumors composed of ECL cells show a loss of MENIN heterozygosity. Type 2 lesions are more common in MEN-1-associated ZES, compared to sporadic ZES, suggesting that the genetic changes in MEN-1 patients may enhance ECL cells susceptibility to the paracrine trophic effects of gastrin [15]. Indeed, a likely pathogenesis of the nearly obstructing ZES-associated malignant/aggressive-like carcinoids seen in our patient.

The role of surgery in MEN-1-associated ZES has historically been aimed at suppression of acid secretion. With the introduction of the aforementioned medical interventions, the role of surgery has shifted toward eradication of primary tumors and control of metastatic spread [5]. Gastrinomas are generally slow-growing tumors and unlikely to metastasize if smaller than 2 cm in size [5]. Although slow growing, patients (60–90%) with larger tumors will present with lymph node involvement, liver, or distant metastasis at the time of diagnosis [5]. Duodenal tumors have a 10% incidence of liver metastases, compared to 50% for pancreatic gastrinomas—with those in the distal pancreas more likely to metastasize to the liver than tumors within the gastrinoma triangle [5, 17]. Metastatic disease, specifically the presence of liver metastasis, significantly worsens prognosis [5, 12]. Importantly, aggressive surgical resection has been shown to improve 5-year survival of patients with metastatic disease from 18–30% to 50–80%, in a later series [18]. While some clinicians may consider avoiding surgery because of the propensity for slow growth, the most common cause of death in these patients is advanced metastatic disease, suggesting a potential benefit for earlier surgical intervention [18]. Typically, resection is reserved for patients with tumor size > 2 cm [6, 19]. Notably, a long-term prospective study of 57 patients that underwent surgery found that among patients with tumors that reached a size of greater than 2.5 cm, 23% of these patients developed liver metastases, further supporting the rationale for an early surgical intervention to prevent disease progression [6, 20].

Surgical resection of locoregional gastrinoma tumors, particularly those larger than 2 cm in size, is recommended except in cases of unresectable disease or extensive liver metastases [6]. Nearly 90% of MEN-1/ZES have lymph node metastasis and require regional lymphadenectomy at the time of surgery. When considering surgical resection of advanced gastrinoma tumors in MEN-1 associated ZES, there are two treatment goals to consider: symptomatic resolution with intent to control/palliate disease and reduction of tumor related mortality. Importantly, an attempt at surgical resection/cytoreduction in a patient with advanced gastrinoma should aim to achieve optimal cytoreduction of > 90% of the gross tumor thought to be resectable on prior imaging studies [4]. This was demonstrated in our patient, whereas initial suboptimal cytoreduction with only enucleation of pancreatic body/tail lesions and regional lymphadenectomy alone did not provide durable disease control (< 29 months) with LAR octreotide but required either concomitant and/or staged (our patient) pancreaticoduodenectomy and gastrectomy to remove the majority (> 90%) of clinically identifiable disease (Fig. 2).

When considering the surgical management of gastrinomas, disease-location influences treatment selection. Treatment of single, small (< 2 cm) duodenal gastrinomas can be managed with transduodenal resection, while multiple locally situated duodenal gastrinomas may require partial resection of the duodenum and regional lymphadenectomy. In the case of multiple/diffuse duodenal and pancreatic head gastrinomas, as in our patient, pancreaticoduodenectomy may be necessary [6]. Furthermore, upon resection of both gastric carcinoid tumors and gastrinomas in MEN-1/ZES patients, it is important to discern that antrectomy alone will have minimal effect on patients with MEN-1/ZES in whom gastrin is ectopically secreted from gastrinoma tumors [5]. In these patients, it is unlikely that a partial gastrectomy will suffice to remove the entirety of the carcinoid tumor  disease burden, particularly in a hypergastrinemic state when incidence of malignant/aggressive gastric carcinoids may exceed 20%. In our case, total gastrectomy was considered, but anatomic limitations and patient related factors precluded complete resection; thus, a near-total gastrectomy was performed.

Aggressive surgical strategies have been recommended for malignant gastrinomas; however, this treatment approach remains controversial [5]. Importantly, in patients with advanced MEN-1-associated ZES, surgical cure is unlikely secondary to clinically occult/undetectable disease which can evade detection and resection [5, 21]. Being that tumors in MEN-1-associated ZES are multicentric and have a long natural disease course, some authors suggest that a procedure as aggressive as a pancreaticoduodenectomy may not be justified because of the significant inherent associated morbidity, including insulin-dependent diabetes mellitus and/or exocrine pancreatic insufficiency [19]. Furthermore, hesitancy may be attributed to the extensive nature of this procedure making reoperation for future lesions problematic [5]. Pancreaticoduodenectomy should be considered with intent for durable disease control in the absence of unresectable metastatic disease when complete gross resection of duodenal and/or pancreatic head gastrinomas can be achieved [4, 5, 22].

In general, surgical outcome studies of patients with MEN-1-associated ZES typically have short follow-up and do not always employ biochemical surrogate serum testing to detect recurrence [5]. In patients with MEN-1-associated ZES, biochemical relapse occurs in greater than 95% of patients within 3 years [23]. Follow-up for MEN-1/ZES patients typically includes an annual fasting gastrin level in addition to imaging and localization studies for patients with elevated gastrin and/or new symptomatology [23]. In our patient, with over 10 years of follow-up and yearly biochemical serum testing, continued suppression of fasting gastrin levels and absence of disease progression persists.

In summary, the favorable outcome described herein in our case report and literature review highlights the potential benefit of optimal tumor cytoreduction followed by LAR octreotide for durable disease control in a patient with MEN-I and ZES. Indeed, the question will remain whether LAR octreotide contributes to durable disease control after optimal cytoreduction of locally advanced gastrinomas and gastric carcinoid tumors. Our case report provides a clinical clue and suggests that it does. To the best of our knowledge, this case highlights the first reported deliberate application of LAR octreotide as post-surgical therapy following optimal cytoreductive surgery for locally advanced disease in a patient with MEN-I and ZES. It is our experience in this patient that LAR octreotide was more effective contributing to long-standing durable disease control only after optimal cytoreduction was performed. This communication represents only a case report with a complex time-line but adds to the growing body of evidence advocating for a more aggressive treatment approach in these patients [4, 5, 22]. Limitations of this case report include the retrospective nature of this review and acquisition of data. Further studies with more patients are required to validate this treatment paradigm.

## Data Availability

Data sharing is not applicable to this article as no datasets were generated or analyzed during the current study.
